# Integrative analyses of morphology, physiology, and transcriptional expression profiling reveal miRNAs involved in culm color in bamboo

**DOI:** 10.3389/fpls.2022.992794

**Published:** 2022-09-09

**Authors:** Chenglei Zhu, Yongfeng Lou, Kebin Yang, Yan Liu, Xiaoyan Xiao, Ziyang Li, Dong Guo, Huayu Sun, Zhimin Gao

**Affiliations:** ^1^Key Laboratory of National Forestry and Grassland Administration/Beijing for Bamboo and Rattan Science and Technology, Beijing, China; ^2^Institute of Gene Science and Industrialization for Bamboo and Rattan Resources, International Center for Bamboo and Rattan, Beijing, China; ^3^Jiangxi Provincial Key Laboratory of Plant Biotechnology, Jiangxi Academy of Forestry, Nanchang, China

**Keywords:** *Phyllostachys vivax* cv. Aureocaulis, culm color, de-domestication, miRNA-mRNA networks, comparative transcriptome

## Abstract

Culm color variation is an interesting phenomenon that contributes to the breeding of new varieties of ornamental plants during domestication. De-domesticated variation is considered ideal for identifying and interpreting the molecular mechanisms of plant mutations. However, the variation in culm color of bamboo remains unknown. In the present study, yellow and green culms generated from the same rhizome of *Phyllostachys vivax* cv. Aureocaulis (*P. vivax*) were used to elucidate the molecular mechanism of culm color formation. Phenotypic and physiological data showed that environmental suitability was higher in green culms than in yellow culms. High-throughput sequencing analysis showed 295 differentially expressed genes (DEGs) and 22 differentially expressed miRNAs (DEMs) in two different colored bamboo culms. There were 103 DEM-DEG interaction pairs, of which a representative “miRNA-mRNA” regulatory module involved in photosynthesis and pigment metabolism was formed by 14 DEM-DEG pairs. The interaction of the three key pairs was validated by qPCR and dual-luciferase assays. This study provides new insights into the molecular mechanism of miRNAs involved in *P. vivax* culm color formation, which provides evidence for plant de-domestication and is helpful for revealing the evolutionary mechanism of bamboo.

## Introduction

Bamboo belongs to the grass family (Poaceae), and is one of the most popular ornamental plants in China having a high socioeconomic and cultural value. The consumption, use, and appreciation of bamboo are traditional Chinese habits, and an important part of Chinese culture. Bamboos have different colors in different organs, such as green, yellow, and purple culms, yellow and green leaves, yellow and purple flowers, etc., among which colored culms are the most important ornamental traits. Since flowering period intervals have a wide range, extending up to > 100 years (Zhao et al., 2013), bamboos predominantly propagate by producing culms from the maternal plant; therefore, selective breeding is the main way to obtain new varieties. Numerous colored-culm ornamental bamboo varieties have been obtained through selective breeding, such as *Phyllostachys vivax* cv. Aureocaulis (*P. vivax*), *P*. *nigra, P*. *violascens, P*. *glauca* f. *yunzhu*, and *Pseudosasa japonica*. Among them, the yellow bamboo of *P*. *vivax* (CYP) is a variety that naturally mutated from *P*. *vivax* f. *huangwenzhu* in 1985, and is characterized by 1–2 thin green stripes on the yellow culms that are different from the original variant (Ma et al., 2014). After nearly 40 years of cultivation, a de-domesticated green mutant (DGM) appeared in the cloned CYP bamboo plants, which has green culm internodes with light yellow groove similar to the prototype of *P*. *vivax* f. *huangwenzhu*. De-domestication is a unique evolutionary process during which crops re-acquire wild-like traits to survive (Jiang et al., 2019). De-domestication studies have expanded our understanding of the complexity of crop evolution, molecular design breeding, and sustainable development of agriculture (Wu et al., 2021).

Variation in the color of bamboo culms has attracted much attention. The suppression subtractive hybridization (SSH) method has been used to identify the genes involved in color variation in bamboo. The results of SSH between green and albino leaves revealed 1,062 and 1,004 differentially expressed transcripts (ESTs) obtained from the forward and reverse SSH libraries of *Ps*. *japonica*, in which 59 ESTs were identified having potential roles in leaf color formation (Yang et al., 2015a). Integrating the phenotype and microscopic observation with the SSH result of culms in *P*. *vivax*, seven ESTs were obtained with higher levels, which were believed to play a role in this color variation (Xia et al., 2015). *PeMPEC* encodes the magnesium-protoporphyrin IX monomethyl ester cyclase in *P. edulis*, and is a well-characterized, an essential enzyme in the biosynthesis of chlorophyll (Yang et al., 2015b). Inter-conversions of violaxanthin, antheraxanthin, and zeaxanthin in *P. edulis* using violaxanthin de-epoxidase (VDE) and zeaxanthin epoxidase were validated (Gao et al., 2013; Lou et al., 2017). With the development of high-throughput sequencing technology, multi-omics has become increasingly important in the study of plant color. Metabolomic and transcriptomic strategies have been used to reveal the underlying variation mechanisms in the internode color of *P*. *violascens* cv. Viridisulcata, from which 81 metabolites and 424 differentially expressed genes (DEGs) were identified. Prunin is a flavanone and rhoifolin, which was discovered to be present at a high level in the culm; *PvGL, PvUF7GT*, and *PvC12RT1*, were involved in prunin or rhoifolin biosynthesis and showed high transcript levels (Wei et al., 2021). RNA-seq technology was applied to investigate the culm color variation of *Bambusa oldhamii*, and 449 key DEGs and 21 differentially expressed transcription factors (TF) were discovered by comparing culm skin samples of *B. oldhamii* and *B. oldhamii* f. *revolute*. Further investigation indicated that *PTAL* is a crucial gene in phenylalanine metabolism and phenylpropanoid biosynthesis pathway, and can cause bamboo culm color variation (Jiao et al., 2022).

Chlorophyll, flavonoids and carotenoids are the major pigments that contribute to plant colors (Grotewold, 2006). Chlorophyll catabolism has been extensively studied, and genes encoding numerous catabolic enzymes have been characterized, including chlorophyll b reductase (Kusaba et al., 2007), chlorophyllase (Tsuchiya et al., 1999), phaeophorbide a oxygenase (Pruzinska et al., 2003), red chlorophyll catabolite reductase (Pruzinska et al., 2007), pheophorbidase (Suzuki et al., 2006), and stay-green (Sato et al., 2007). Studies have also been conducted on phytoene synthase in the carotenoid biosynthesis pathway (Zhou et al., 2022), and chalcone synthase (CHS), chalcone isomerase (CHI), flavonol 3-hydroxylase (F3H), dihydroflavonol-4-reductase (DFR), leucoanthocyanidin dioxygenase, and anthocyanidin reductase (ANR) in the proanthocyanidins biosynthesis pathway (Xu et al., 2015). Several TFs of the bZIP, NAC, bHLH, WRKY, and MYB families are involved in chlorophyll, flavonoid, and carotenoids biosynthesis (Hu et al., 2019; Yang et al., 2021c; Gan et al., 2022; Li et al., 2022a; Yu et al., 2022). MiRNAs have been reported to play important roles in chlorophyll and flavonoid biosynthesis (Gou et al., 2011; Li et al., 2018; Tirumalai et al., 2019). Although several structural genes and TFs involved in the syntheses of chlorophyll, lutein and anthocyanin are known to affect the color of plants, and some information exists of the bamboos with different colors, the mechanism of miRNAs involved in culm color changes during the evolutionary process remains unclear. In this study, we employed the naturally mutated culms of *P. vivax* to identify the phenotypic and physiological changes and reveal the genetic elements involved in controlling culm color. These findings provide insights into the expression profiles of important miRNA-mRNA interaction pairs in the representative regulatory module related to photosynthesis and pigment metabolism. Moreover, the evidences presented here offer an entry point to understanding the process of color variation in greater detail.

## Materials and methods

### Measurement of plant phenotypic and physiological parameters

Yellow and green bamboos of *P. vivax* (CYP and DGM) were harvested from a bamboo garden at the Jiangxi Academy of Forestry Sciences, Nanchang, Jiangxi Province, China, in April 2020. The bamboo height, under-branch height, diameter at breast height (DBH), internode diameter (ID), and internode wall thickness (IWT) were measured with rulers. An LI-6400 portable photosynthesis measurement system was used to measure the net photosynthetic rate (NPR), stomatal conductance (SC), intercellular CO_2_ concentration and transpiration rate. The 7^th^ and 11^th^ internodes of bamboo shoots with 3.0 m height were selected as the experimental materials. The 7^th^ and 11^th^ yellow or green internodes were designated as Y1 and Y2, or G1 and G2, respectively. Samples were collected for RNA isolation, biochemical analysis, and enzyme activity determination. The chlorophyll a, chlorophyll b, carotenoids, soluble sugars, flavonoids, and anthocyanins concentrations were measured using assay kits (Geruisi, G0613F, G0501F, G0118F, and G0126F, China) according to the manufacturer's protocol. The enzymes activities of ChlG, POR, CAO, and CHI were determined by enzyme-linked immunosorbent assay (ELISA) kits (Ruixinbio, CK1400781PL, CK1401586PL, CK1400630PL, and CK1401449PL, China) with ELISA analytical instrument (Rayto, RT-6100, USA).

### RNA isolation, library construction, transcriptome, small RNA, and degradome sequencing

Total RNA was extracted from four types of samples using the Total RNA Kit (Tianmo, TR205-50, China) for transcriptome; small RNA, and degradome sequencing, and three biological replicates were used. Total RNA was checked for quality and purity using an Agilent Bioanalyzer (Agilent, 2100, USA) and NanoDrop (Thermo, NanoDrop2000, USA), respectively, which were further used for sequencing. Library construction, quality control, and data processing were executed as in previous studies (Yang et al., 2021b; Li et al., 2022c). Transcriptome and small RNA sequencing libraries were generated using the NEBNext^®^ Ultra^™^ RNA Library Prep Kit for Illumina^®^ (NEB, E7530L, USA), and library quality was assessed using an Agilent Bioanalyzer 2100 system. Samples were analyzed on a cBot Cluster Generation System using the TruSeq PE Cluster Kit v3-cBot-HS (Illumina, PE-401-3001, USA), respectively. After cluster generation, the library preparations were sequenced on an Illumina Hiseq 2000 platform. Sequencing data were quality-controlled using FASTQC (BaseSpace Labs, Illumina, USA), from which low-quality data (Q30 < 85% and unknown *N* > 10%) of transcriptome and low-quality data (Length < 15 nt and > 35 nt; unknown *N* > 10%; without 3' adapter sequences) of small RNA were filtered. High-quality clean data were used for sequence assembly and Trinity software (Grabherr et al., 2011) to obtain the unigene library. The degradome was analyzed by CleaveLand program (v.4.0) (Addo-Quaye et al., 2009), and the Oligomap short reading frame calibrator was used to find the mRNAs that matched the degradation group sequence (Berninger et al., 2008). The Needle program in the EMBOSS package (v.6.6.0) was used for scoring analysis according to the plant miRNA/target pairing criteria (Allen et al., 2005). The transcriptome and small RNA sequencing data were deposited in NCBI with the accession numbers of PRJNA839516 (https://dataview.ncbi.nlm.nih.gov/object/PRJNA839516) and PRJNA841040 (https://dataview.ncbi.nlm.nih.gov/object/ PRJNA841040). The detailed information was listed in Supplementary Table S1.

### Identification and annotation of DEGs and DEMs

Bowtie (v.1.2.2) (Langmead et al., 2009) was used to align the reads obtained by sequencing with the unigene library. The value of fragments per kilobase of transcript per million mapped reads (FPKMs) was used to represent the expression abundance of protein-coding transcripts using StringTie (v.1.3.1) software. To compare the expression of genes and miRNAs across the four types of samples between CYP and DGM, differentially expressed genes (DEGs) and miRNAs (DEMs) of the two groups (Y1 vs. G1 and Y2 vs. G2) were analyzed using the DESeq2 R package (v.1.14.1). DESeq2 provided statistical routines for determining the differential expression in digital gene expression data using a model based on negative binomial distribution. The resulting *P* values were adjusted using Benjamini and Hochberg's approach to control the false discovery rate. Genes with fold change (FC) ≥ 2 and *P* ≤ 0.05 were assigned as DEGs. FC1_1-3 and FC2_1-3 indicate the expression ratios of G1_1-3/Y1_1-3 and G2_1~3/Y2_1~3, respectively. MiRNAs with FC ≥ 1.5; *P* ≤ 0.05 were assigned as DEMs.

### Functional prediction of DEGs

To identify the functions of DEGs, Gene Ontology (GO) enrichment analysis was implemented using the GOseq R packages (v.1.46.0) based on the Kolmogorov-Smirnov test and Wallenius non-central hyper-geometric distribution. KEGG is a database resource for understanding high-level functions and utilities of biological systems (Kanehisa et al., 2008), such as cells, organisms, and ecosystems, from molecular-level information, especially large-scale molecular datasets generated by genome sequencing and other high-throughput experimental technologies (http://www.genome.jp/kegg/). The KOBAS software (v.3.0.3) was used to test the statistical enrichment in the KEGG pathways (Mao et al., 2005).

### Prediction of miRNA binding sites in DEGs and regulatory network construction

The numerous miRNA-binding sites in the mRNA sequences were predicted using TargetFinder (v.1.1.3) software. Interactions between miRNAs and mRNAs were further divided into coherent or non-coherent pairs based on their expression patterns. The interaction pairs of DEMs and DEGs were used to further analyze the miRNAs function. The regulatory networks of DEMs and DEGs were visualized using the Cytoscape (v.3.7.1) software.

### Validation of the DEGs and DEMs

First-strand complementary DNA (cDNA) was synthesized from the total RNA of four samples using a PrimeScript RT Reagent kit (Takara, RR037A, Japan). Quantitative real*-*time PCR (qPCR) of mRNAs was conducted using the Roche Light Cycler 480 SYBR^®^ Green I Master kit (Roche, 04887352001, Germany) with specific primers (Supplementary Table S2). *ACT2-1* was used as the internal control (Xia et al., 2015). MiRNA first-strand cDNA was synthesized by miRNA First Strand cDNA Synthesis Kit (Aidlab, PC4801, China), and qPCR of miRNAs was conducted using the miRNA Universal SYBR^®^ qPCR Master Mix (Aidlab, PC4901, China) with specific primers (Supplementary Table S2). *U6* snRNA was used as an internal control (Ding et al., 2011). Three independent experiments were performed, and each experiment was repeated thrice.

Wild-type (WT) of two miRNAs and three 200 bp long genes, including the predicted splicing sites, flanking sequence, and mutant type (MUT) after site-directed mutation of WT target sites, were synthesized artificially (Ruibiotech, Beijing, China). These were then cloned into the pmirGLO vector (GeneCreate, Wuhan, China). After confirmation by sequencing, MUT and WT vectors were co-transfected with negative control (NC) and miRNA mimics, respectively into 293T cells (Li et al., 2022c). The relative luciferase activity was measured after 48 h transfection using Luciferase reporter assay kit (Beyotime, RG027, China) and then normalized to Renilla luciferase activity. The proportion of Firefly/Renilla luciferase activity in each cell line was used to the quantify outcomes. Three independent experiments were performed. The NC, miRNA mimics, WT and MUT sequences information in plasmids were listed in Supplementary Table S3.

### Statistical analysis

All statistical data were assessed for significant differences using Duncan's multiple range test using the SPSS statistics package (v.21.0). All data are presented as mean ± standard deviation (SD) of at least three replicates. Graphs were constructed and verified using Origin (v.8.0) and Adobe Illustrator CC (v.2018).

## Results

### Phenotype and physiological characteristics of CYP and DGM culms

**Figure 1:**
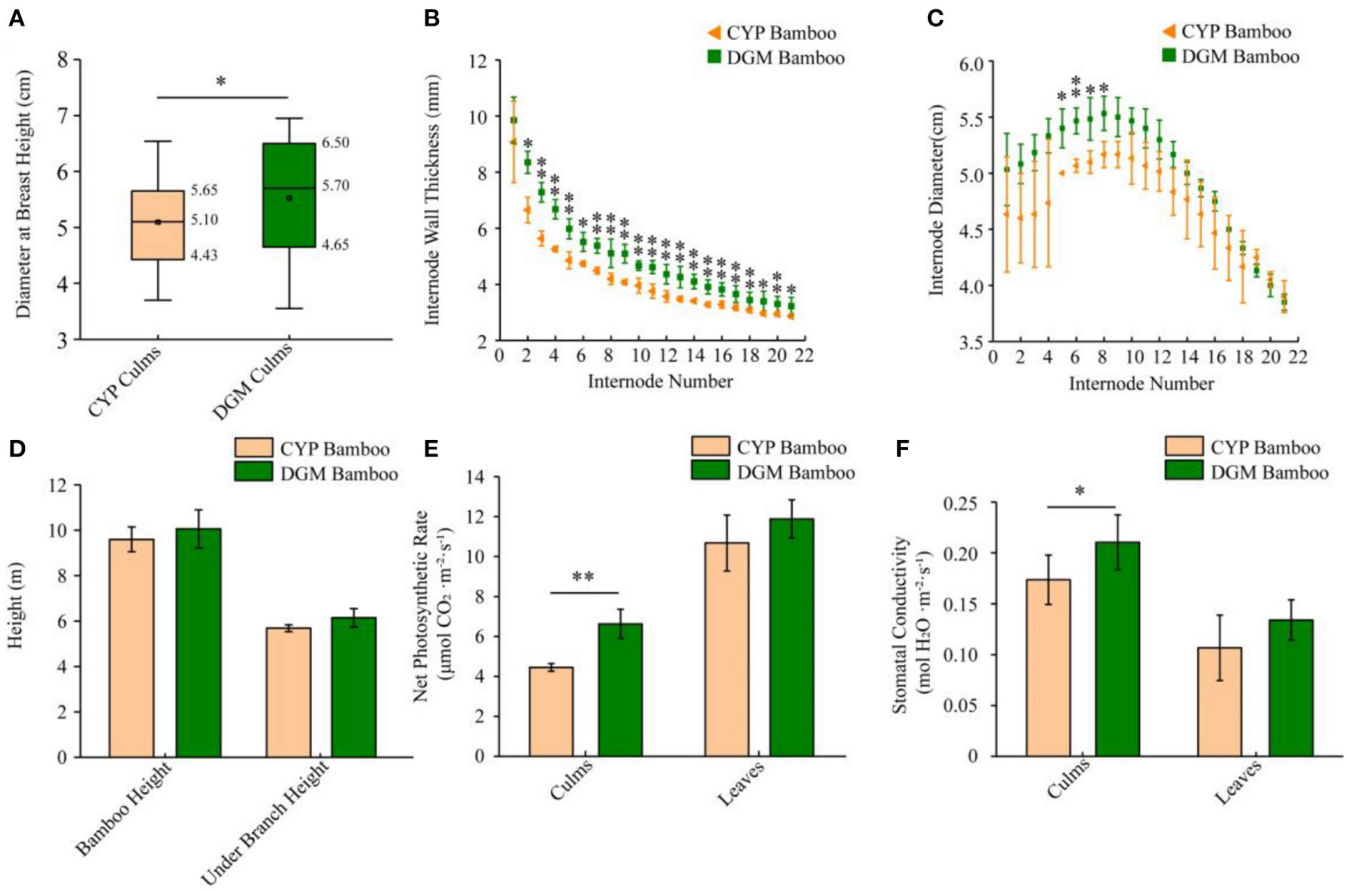
Phenotypic characteristics of CYP and DGM bamboos of *P. vivax*. **(A)** Average DBH of CYP and DGM culms. **(B)** Average IWT of one year old culms under branch. **(C)** Average ID of one year old culms under branch. **(D)** Average bamboo height and under branch height of one year old CYP and DGM culms. **(E)** Net photosynthetic rate of culms and leaves of CYP and DGM bamboos. **(F)** Stomatal conductance of culms and leaves of CYP and DGM bamboos. Data is presented as the mean ± standard deviation. Statistical analyses were performed by student's *t*-test. ^*^0.01 ≤ *p* ≤ 0.05 and ^**^p < 0.01.

*P. vivax* is characterized by yellow culms, which are considered ornamental plants and are extensively planted in gardens. The differences between yellow and green culms generated from the same rhizome of *P. vivax* were investigated in this study. Compared to CYP, one-year-old DGM bamboo displayed a wider range of phenotypic variations. The DBH and IWT of DGM culms were far larger than those of CYP culms, and the 5^th^, 6^th^, 7^th^, and 8^th^ internode IDs of DGM were much longer than those of CYP culms (Figures 1A–C). Furthermore, the whole height, under-branch height and internode length of two bamboo culms were not significantly different (Figure 1D, Supplementary Figure S1A). Photosynthetic physiology measurement revealed that the NPR and SC of DGM culms were higher than those of CYP culms (Figures 1E,F). Nevertheless, no significant differences were observed in intercellular CO_2_ concentrations and transpiration rates between the two bamboo culms, as well as in the NPR and SC of the two bamboo leaves (Supplementary Figures S1B,C).

**Figure 2:**
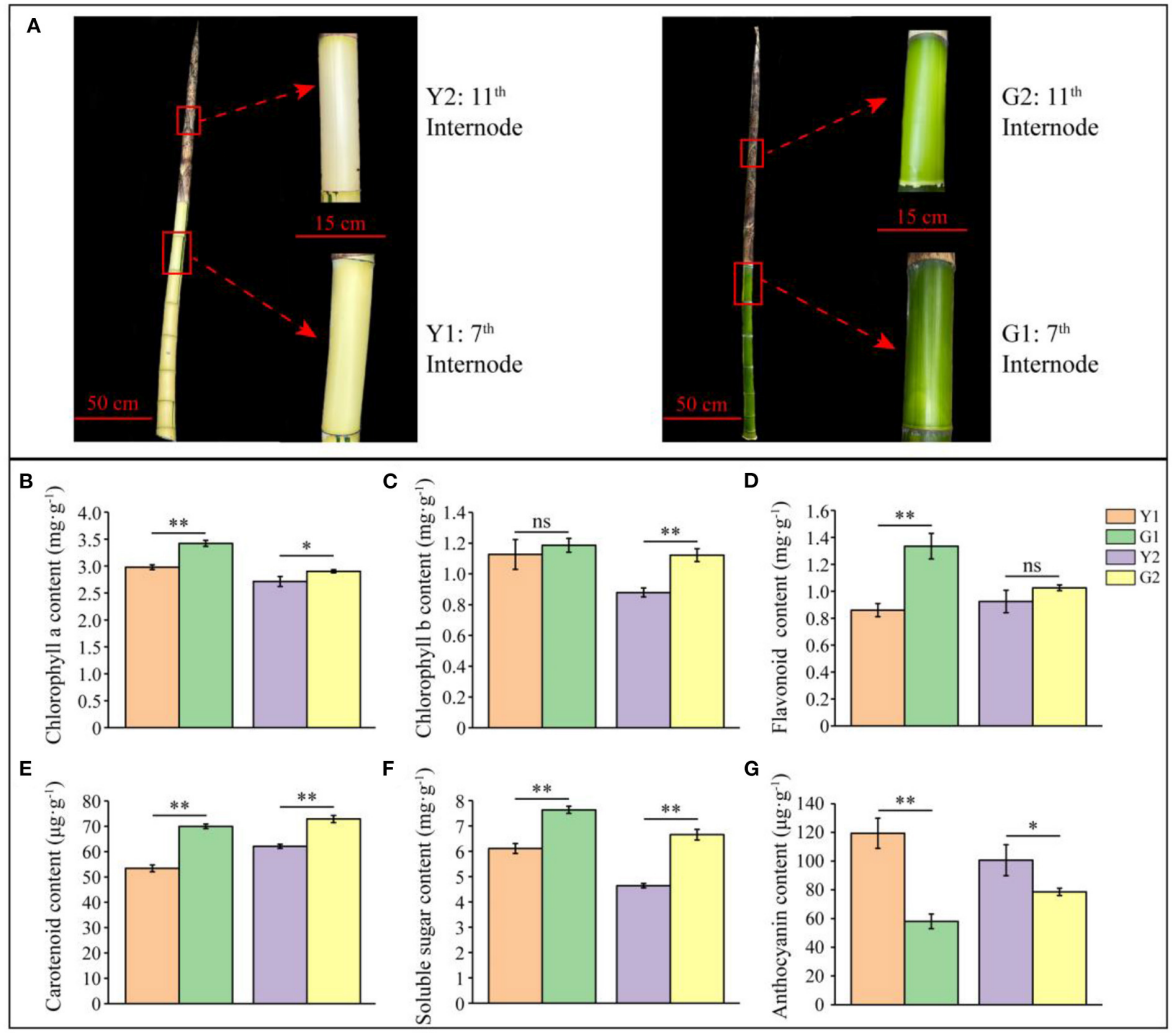
Phenotype and biochemical characteristics of CYP and DGM shoots of *P. vivax*. **(A)** Shoot phenotype characteristics and representative internode of CYP and DGM culms. **(B–G)** Measurement of chlorophyll a, chlorophyll b, flavonoid, carotenoid, soluble sugar, and anthocyanin contents of CYP and DGM. Data is presented as the mean ± standard deviation. Statistical analyses were performed by student's *t*-test. ^*^0.01 ≤ *p* ≤ 0.05; ^**^p < 0.01; ns: *p* > 0.05.

Furthermore, two representative shoot internodes (7^th^ and 11^th^) from CYP and DGM culms were selected, in which shoot sheaths of the 7^th^ shoot internodes fell off and those of the 11^th^ shoot internodes were still intact (Figure 2A, Supplementary Figure S2). External characteristics of the CYP and DGM shoots were significantly different. Thus, two comparisons (Y1 vs. G1 and Y2 vs. G2) from four samples (the 7^th^ and 11^th^ internodes of CYP and DGM shoots: Y1, G1, Y2, and G2) were used for further analysis. Concentrations of the six biochemical components were measured using the kit methods (Figures 2B–G). Compared with the CYP internodes, chlorophyll a, chlorophyll b, flavonoid, carotenoid, and soluble sugar concentrations were higher, while anthocyanin concentrations were lower in DGM, among which chlorophyll a, carotenoid, and soluble sugar concentrations were significantly different in the two comparisons of Y1 vs. G1 and Y2 vs. G2. Chlorophyll b concentration was significantly different only in Y2 vs. G2, whereas no significant difference was observed in Y1 vs. G1. Flavonoid content was significantly different in Y1 vs. G1, but no significant was observed difference in Y2 vs. G2. These results indicate that DGM with green culms is a de-domesticated variety of CYP.

### Transcriptomic changes in two bamboo culms with different colors

To determine the discrepancy between the two different colored culms of CYP and DGM, 12 samples were sequenced using RNA-Seq technology, from which 30.21 million raw reads (90.48 Gb) were generated. The GC content was ~54.05%, and 95.14% (28.75 million) high-quality reads were processed for further analysis after quality control (Supplementary Table S4). A total of 89,874 unigenes were obtained after assembly, with an average unigene length of 816.27 bp, among which 19,716 were >1,000 bp in length (Supplementary Table S5). Furthermore, 32,708 unigenes were annotated by comparison with NR, Swiss-Prot, KEGG, COG, KOG, GO, and Pfam databases (Supplementary Table S6).

**Figure 3:**
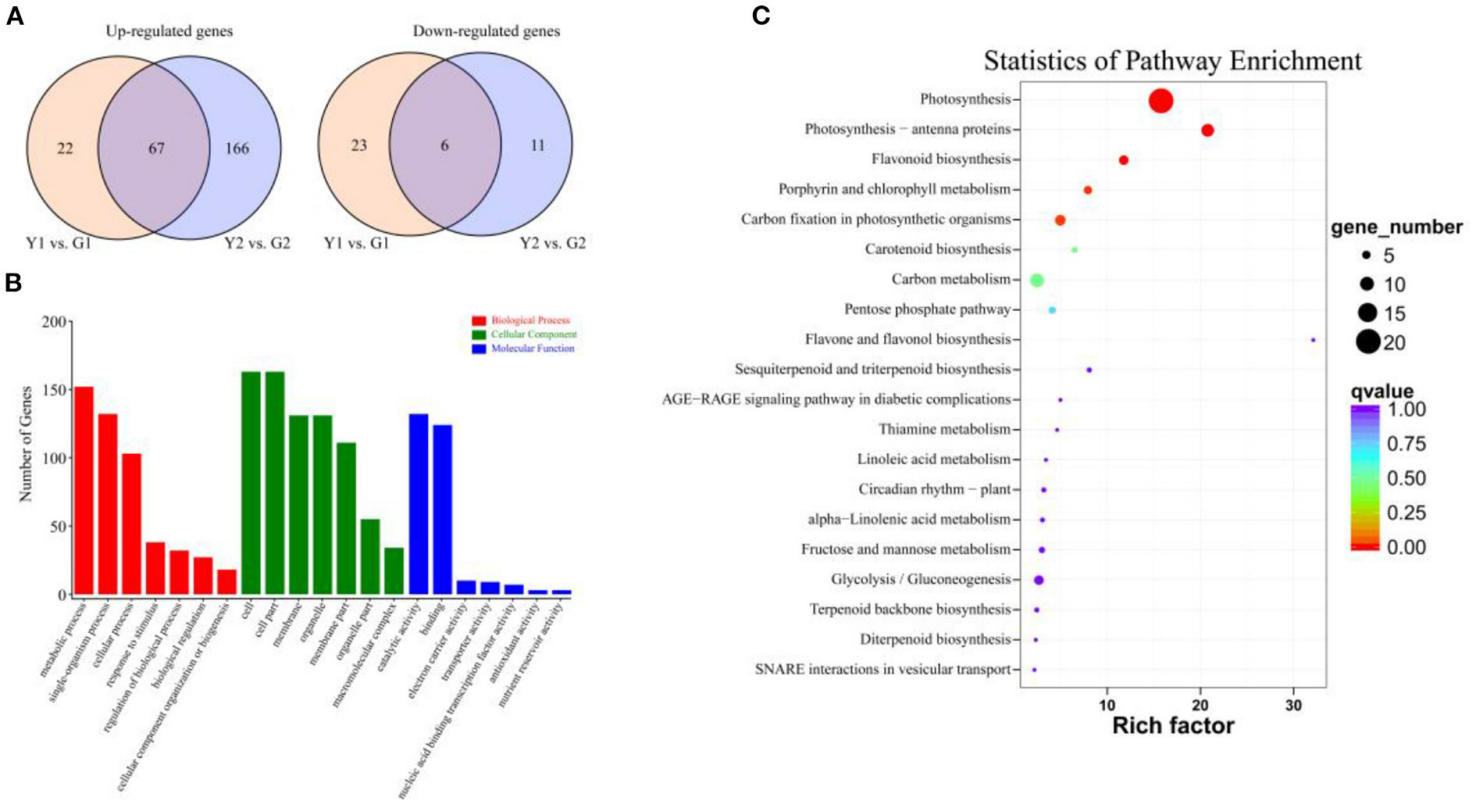
The number of DEGs identified by comparisons and enrichment analysis. **(A)** Venn diagrams show the number of up- and down-regulated DEGs in two comparisons. Yellow and blue circle indicates Y1 vs. G1 and Y2 vs. G2 comparisons, respectively. **(B)** GO enrichment analysis of DEGs, including molecular function (blue bar-graph), cellular component (green bar-graph), and biological process (red bar-graph). **(C)** KEGG enrichment analysis of DEGs.

A total of 295 DEGs were detected in at least one pairwise comparison (Y1 vs. G1 or Y2 vs. G2), among which 255 were up-regulated, and 40 were down-regulated, the number of DEGs (250) in Y2 vs. G2 was much higher than that in Y1 vs. G1 (118). A total of 250 DEGs were identified in the Y2 vs. G2 comparison, with 233 up-regulated and 17 down-regulated genes. Similarly, 118 DEGs were identified in the Y1 vs. G1 comparison, with 89 up-regulated and 29 down-regulated DEGs (Figure 3A).

GO analysis was used to explore the biological functions of DEGs between the different colored culms of CYP and DGM (Figure 3B). The DEGs covered three GO categories: “biological progress (BP),” “cellular component (CC),” and “molecular function (MF)”. Within the BP category, DEGs were mainly involved in “Metabolic process,” “Single-organism process,” and “Cellular process.” As for the GO terms of CC, the DEGs were mainly included “Cell,” “Cell part,” “Membrane,” “Organelle,” and “Membrane part”; In the MF categories, the DEGs were mainly enriched in “Catalytic activity” and “Binding.” The KEGG pathway enrichment analysis was performed to further characterize the DEG-associated pathways (Figure 3C). These DEGs were involved in 43 KEGG pathways, among which photosynthesis (ko00195) and photosynthesis-antenna proteins (ko00196) attained the top positions in two comparisons. Flavonoid biosynthesis, porphyrin and chlorophyll metabolism pathways, which are important metabolic pathways for plant pigment synthesis, were also significantly enriched. Furthermore, 11 DEGs were classified into seven TF families, including MYB, MADS and Homebox, based on Pfam annotation.

### Identification of DEGs related to photosynthesis metabolism

**Figure 4:**
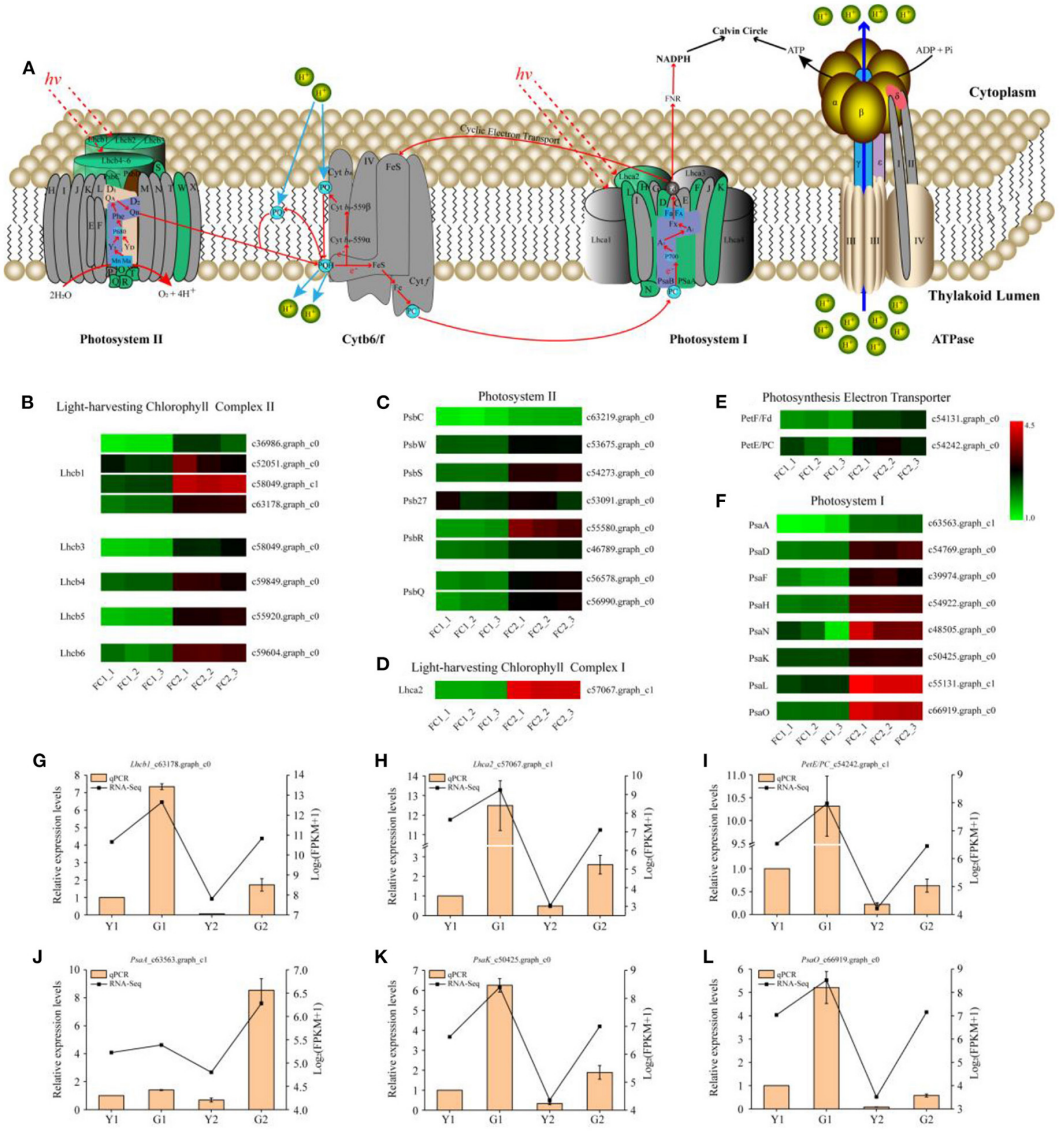
DEGs predicted to be involved in photosynthesis metabolism. **(A)** Diagram of electron transport pathway in photosynthesis. **(B–F)** DEGs involved in light-harvesting chlorophyll complex II, photosystem II, light-harvesting chlorophyll complex I, photosynthesis electron transporter, and photosystem II, respectively. Lhcb light-harvesting complex II chlorophyll a/b binding protein, PsbC photosystem II CP43 chlorophyll apoprotein, PsbW photosystem II W protein, PsbS photosystem II 22 kDa protein, Psb27 photosystem II 27 protein, PsbR photosystem II 10 kDa protein, PsbQ photosystem II oxygen-evolving enhancer protein, Lhca light-harvesting complex I chlorophyll a/b binding protein, PetF/Fd ferredoxin, PetE/PC plastocyanin, PsaA photosystem I P700 chlorophyll a apoprotein A1, PsaD photosystem I reaction center subunit II, PsaF photosystem I subunit III, PsaH photosystem I reaction center subunit VI, PsaN photosystem I subunit N, PsaK photosystem I P700 chlorophyll a apoprotein A1, PsaL photosystem I reaction center subunit XI, PsaO photosystem I subunit O. **(G–L)** Relative expression of six selected genes validated by qPCR. Data is presented as the mean ± standard deviation.

As photosynthesis and photosynthesis-antenna protein pathways are enriched in DEGs, their functional annotations were further characterized. The proteins encoded by the DEGs were located in the chloroplast and annotated to function as photosystem I (PSI) and photosystem II (PSII) components, photosynthetic electron transport, and photosynthesis-antenna proteins (KEGG map: 00062; Figure 4A). A total of 27 DEGs related to photosynthesis were identified in these comparisons, among which, we focused on the transcriptional levels of genes that are closely related to photosynthetic efficiency. Eighteen genes encoding proteins (Figures 4B–D), which were involved in the reaction center and electron transport in photosynthesis, including the PSI core protein (PsaA), PSI receptor site subunits (PsaD, PsaN), PSI membrane interface subunits (PsaF, PsaL, PsaK, and PsaH), PSII manganese-stabilizing protein (PsbO), PSII 10 kDa protein (PsbR), PSII 6.1 kDa protein (PsbW), PSII repair protein Psb27-H1 (Psb27), plastocyanin (PC), and ferredoxin (Fd) of photosynthetic electron transport (PetE and PetF), were up-regulated. Moreover, all annotated DEGs of light-harvesting Chl a/b binding protein complex I and II (Lhcs), namely, one gene encoding the Chl a/b binding protein complex I (Lhca2) and eight genes encoding Chl a/b binding protein complex II (Lhcb1, Lhcb3, Lhcb4, Lhcb5, and Lhcb6) were up-regulated during the greening process of *P. vivax* (Figures 4E,F). Additionally, qPCR results showed that the expression patterns of the six genes were consistent with the RNA-seq results (Figures 4G–L).

### Identification of DEGs related to pigment metabolism

#### DEGs in porphyrin and chlorophyll metabolism

**Figure 5:**
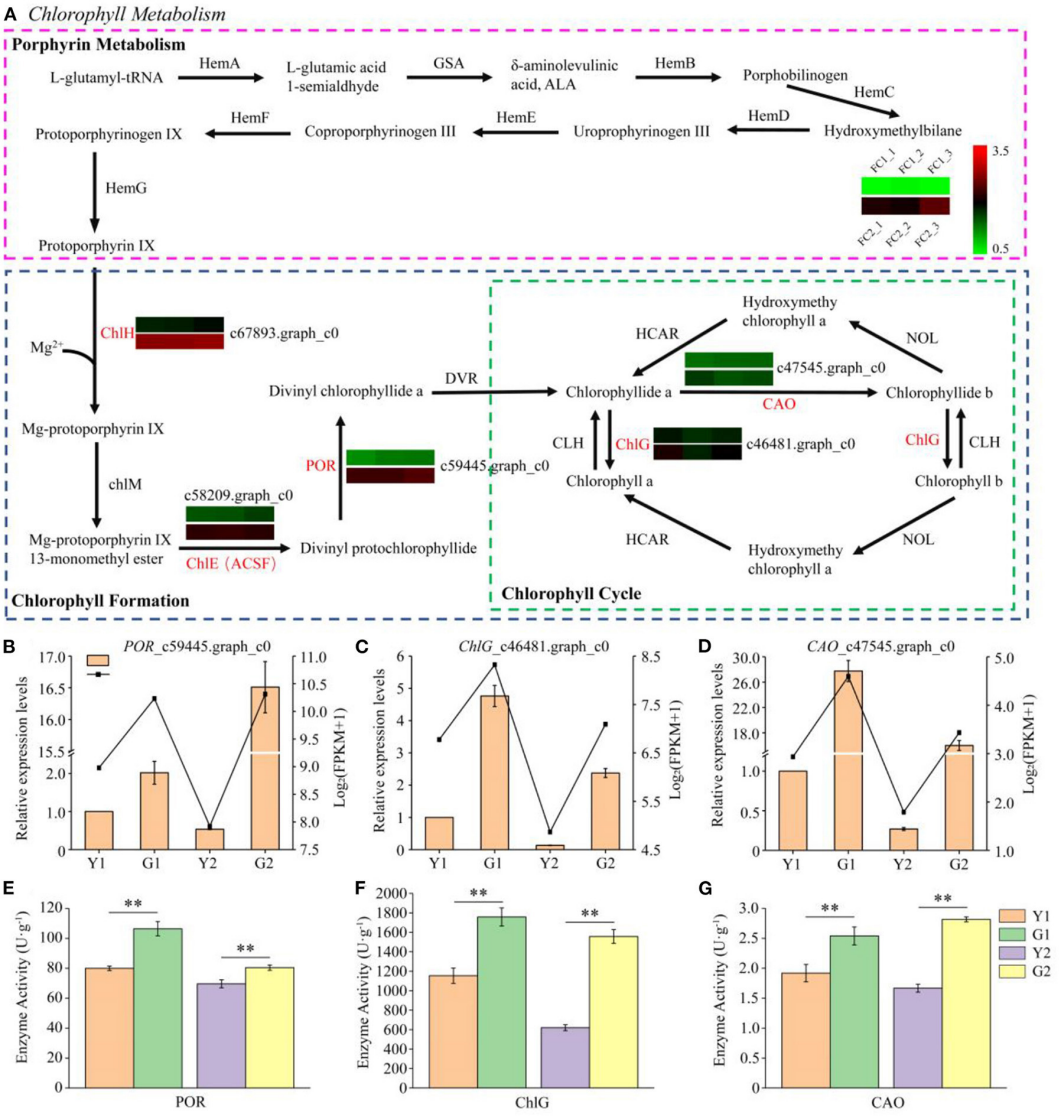
Transcriptional profiling of DEGs involved in porphyrin and chlorophyll metabolism. **(A)** DEGs involved in porphyrin and chlorophyll metabolism. HemA glutamyl tRNA reductase, GSA glutamate 1-semialdehyde aminotransferase, HemB porphobilinogen synthase, HemC hydroxymethylbilane synthase, HemD uroporphyrinogen III synthase, HemE uroporphyrinogen III decarboxylase, HemF coproporphyrinogen oxidative decarboxylase, HemG protoporphyrinogen synthase, ChlH magnesium chelatase H subunit, ChlM magnesium-protoporphyrin IX methyltransferase, ChlE (ACSF) magnesium-protoporphyrin IX monomethyl ester (oxidative) cyclase, POR protochlorophyllide reductase, DVR 3,8-divinyl chlorophyllide a 8-vinyl reductase, CAO chlorophyllide a oxygenase, ChlG chlorophyll synthase. **(B–D)** Relative expression of three selected genes validated by qPCR. **(E–G)** Determination of POR, ChlG and CAO enzyme activities. Data is presented as the mean ± standard deviation. Statistical analyses were performed by student's *t*-test. ^**^p < 0.01.

Eighteen enzyme genes were involved in porphyrin and chlorophyll metabolism. Eight, five and five enzymes were involved in porphyrin metabolism, chlorophyll formation, and the chlorophyll cycle, respectively (Figure 5A). Five DEGs, the key candidate genes encoding enzymes, were associated with porphyrin and chlorophyll metabolism. In the greening process of *P. vivax, ChlH*_c46481.graph_c0, *ChlE*_c67893.graph_c0, and *POR*_c59445.graph_c0 of chlorophyll formation were observed to be significantly up-regulated in the two comparisons, and two genes, *ChlG*_c47545.graph_c0, and *CAO*_c58209.graph_c0, in the chlorophyll cycle were up-regulated; however, no DEGs were identified in porphyrin metabolism. Furthermore, qPCR results showed that the expression patterns of *POR*_c59445.graph_c0, *ChlG*_c47545.graph_c0, and *CAO*_c58209.graph_c0 were consistent with the RNA-seq results (Figures 5B–D). Furthermore, the activities of enzymes encoded by these three genes were measured using ELISA kits, and were significantly higher in G1 and G2 samples than those in Y1 and Y2 samples, respectively (Figures 5E–G).

#### DEGs in flavonoid biosynthesis metabolism

**Figure 6:**
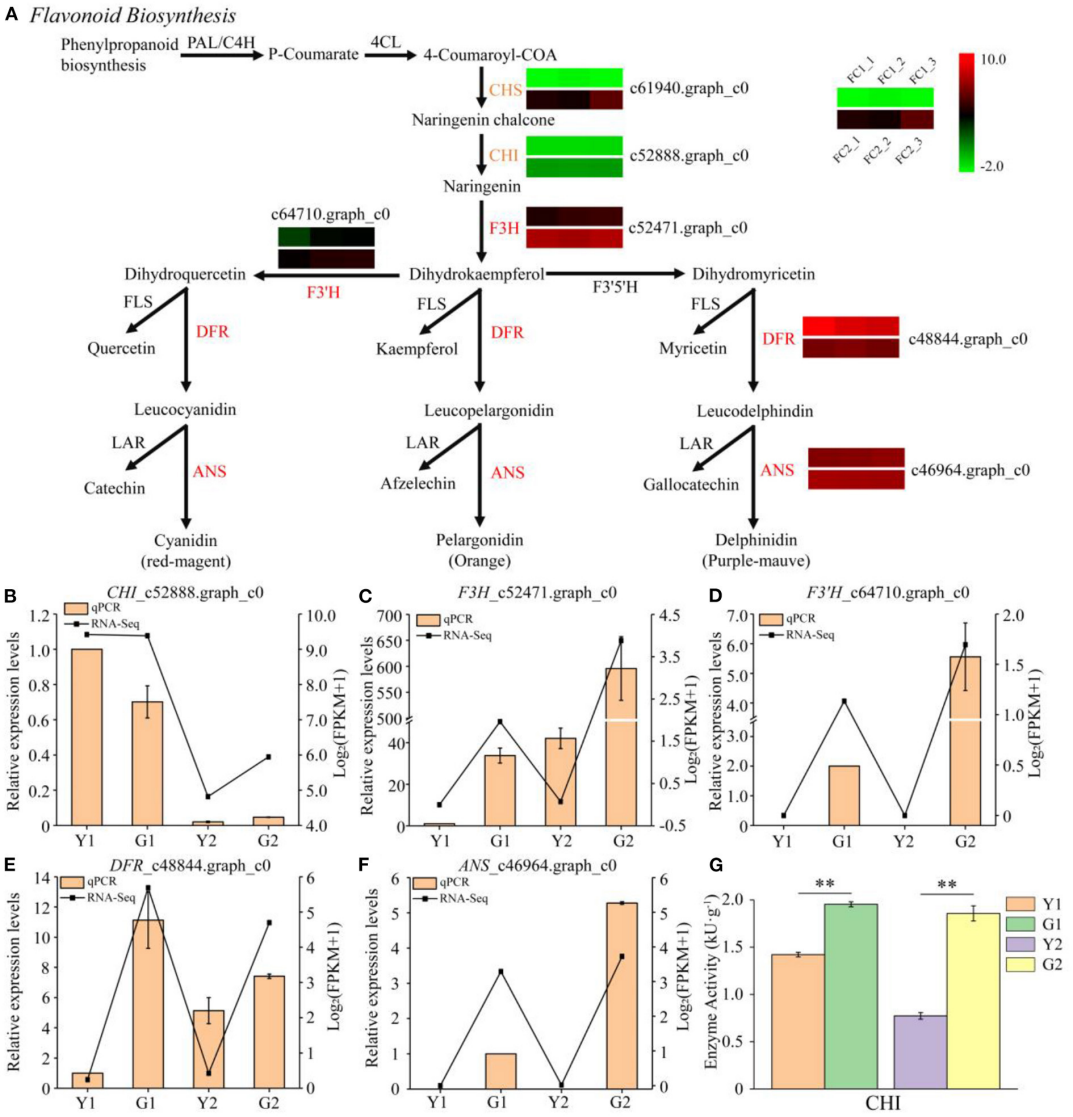
Transcriptional profiling of DEGs involved in flavonoid biosynthesis. **(A)** DEGs involved in flavonoid biosynthesis. PAL phenylalanine ammonia-lyase, 4CL 4-coumarate CoA ligase, CHS chalcone synthase, CHI chalcone isomerase, F3H flavanone 3-hydroxylase, F3′5′H flavonoid 3′5′-hydroxylase, F3′H flavonoid 3′-hydroxylase, DFR dihydroflavonol 4-reductase, ANS anthocyanidin synthase, LAR leucocyanidin reductase, FLS flavonol synthase. **(B–F)** Relative expression of five selected genes validated by qPCR. Error bars indicate standard deviation (SD) of three technical repeats. **(G)** Determination of CHI enzyme activity. Data is presented as the mean ± standard deviation. Statistical analyses were performed by student's *t*-test, ^**^p < 0.01.

Six DEGs were annotated in the flavonoid biosynthesis pathway (Figure 6A). *F3H*_c52471.graph_c0, *DFR* _c48844.graph_c0, and *ANS*_c46964.graph_c0 were significantly up-regulated during the greening process in Y1 vs. G1. In addition, three other genes, *CHS*_c61940.graph_c0, *CHI*_c52888.graph_c0, and *F3*′*H*_c64710.graph_c0, were up-regulated during the greening process in Y2 vs. G2. However, no down-regulated DEGs were identified in the flavonoid biosynthetic pathway. Five key DEGs were selected for the qPCR analysis (Figures 6B–F). qPCR results showed that the expression levels of *F3*′*H*_c64710.graph_c0 and *ANS*_c46964.graph_c0 were not detected in Y1 and Y2 samples, but were highly expressed in G1 and G2 samples. The expression levels of *F3H*_c52471.graph_c0 and *DFR* _c48844.graph_c0 were higher in G1 and G2 than in Y1 and Y2, respectively. Meanwhile, the changed trends of CHI enzyme activity were different from the expression of *CHI*_c52888.graph_c0 in Y1 and G1 samples but similar in Y2 and G2 samples (Figures 6B,G).

### Identification and expression of miRNAs in CYP and DGM culms

**Figure 7:**
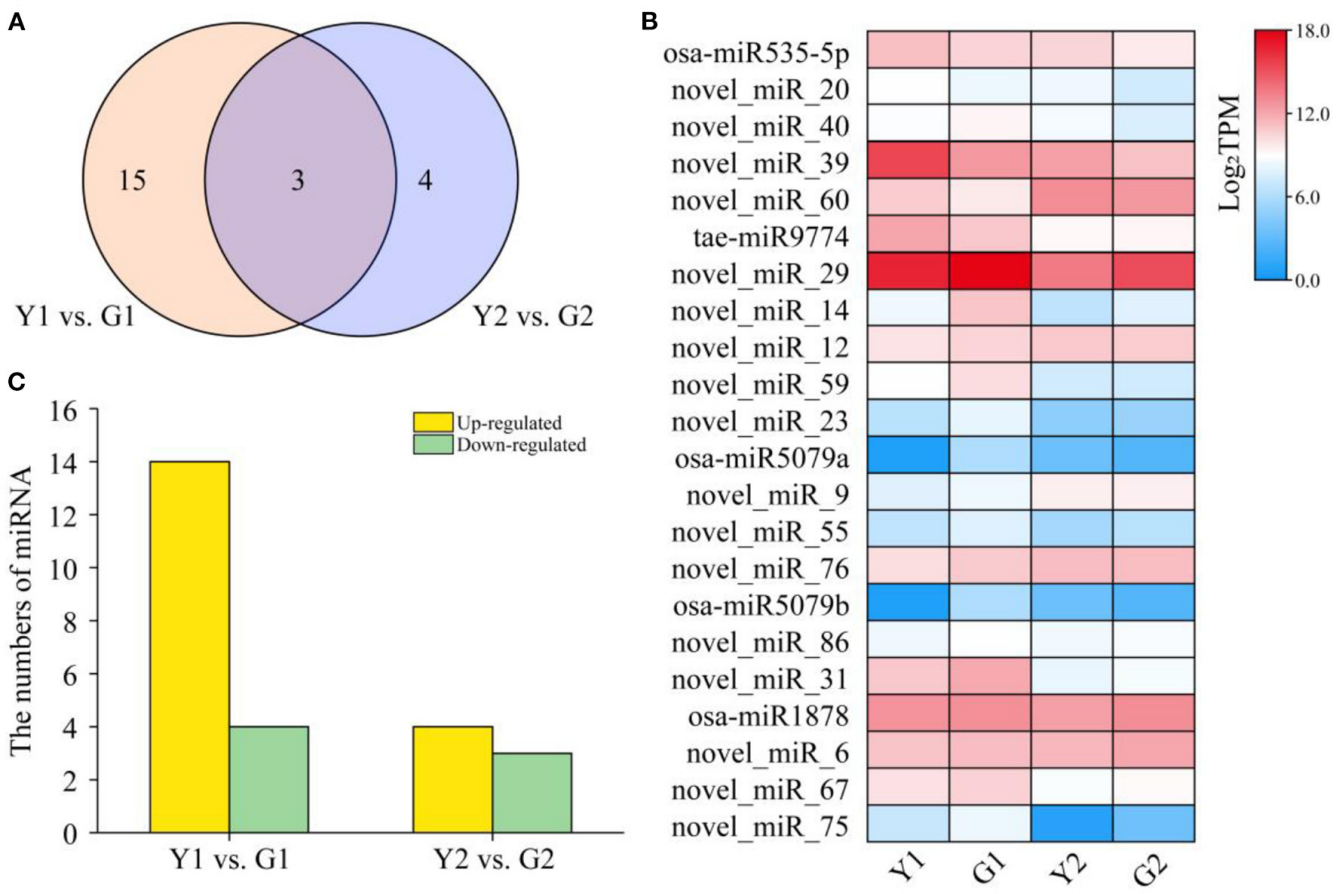
Different expression analysis of miRNAs in the greening process of *P. vivax*. **(A)** Venn diagram of miRNA distribution in two comparisons, respectively. **(B)** Heatmap of expression of all DEMs in four samples. **(C)** Specific expression distribution of DEMs in two comparisons.

To identify the miRNAs involved in culm greening of CYP and DGM, 12 small RNA libraries were constructed and sequenced. A total of 24.08 million clean reads were obtained after filtering low-quality reads. It was observed that 24 nt small RNAs (37.39%) were the most abundant, followed by 21 nt (33.04%), 22 nt (10.43%), and 23 nt small RNAs (6.96%). The filtered reads were searched for miRNAs in the miRbase database. In total, 115 miRNAs were identified, of which 27 were matched with known miRNAs and 88 were predicted to be novel. Only four of the 35 families had more than three members, among which MiR171_1 was the largest family with nine members, followed by MiR160 (6), Pvi-miR396 (3), and MiR535 (3). In contrast, the remaining 32 families had only one or two members, such as MiR159, MiR164, MiR162_2, and MiR169_1 (Supplementary Table S7). Expression patterns of the identified miRNAs were further analyzed to detect those involved in the greening process of culms. A total of 22 DEMs including five known miRNAs and 17 novel miRNAs were found across two comparisons (Figure 7A), and the expression of DEMs in each group was also displayed (Figure 7B). Among them, four down-regulated and 14 up-regulated miRNAs were found in the Y1 vs. G1 comparison, and three down-regulated and four up-regulated miRNAs were found in the Y2 vs. G2 comparison (Figure 7C). These identified DEMs may have specific functions in the greening process of *P. vivax*.

### Targets identification of DEMs *via in silico* and degradome analyses

**Figure 8:**
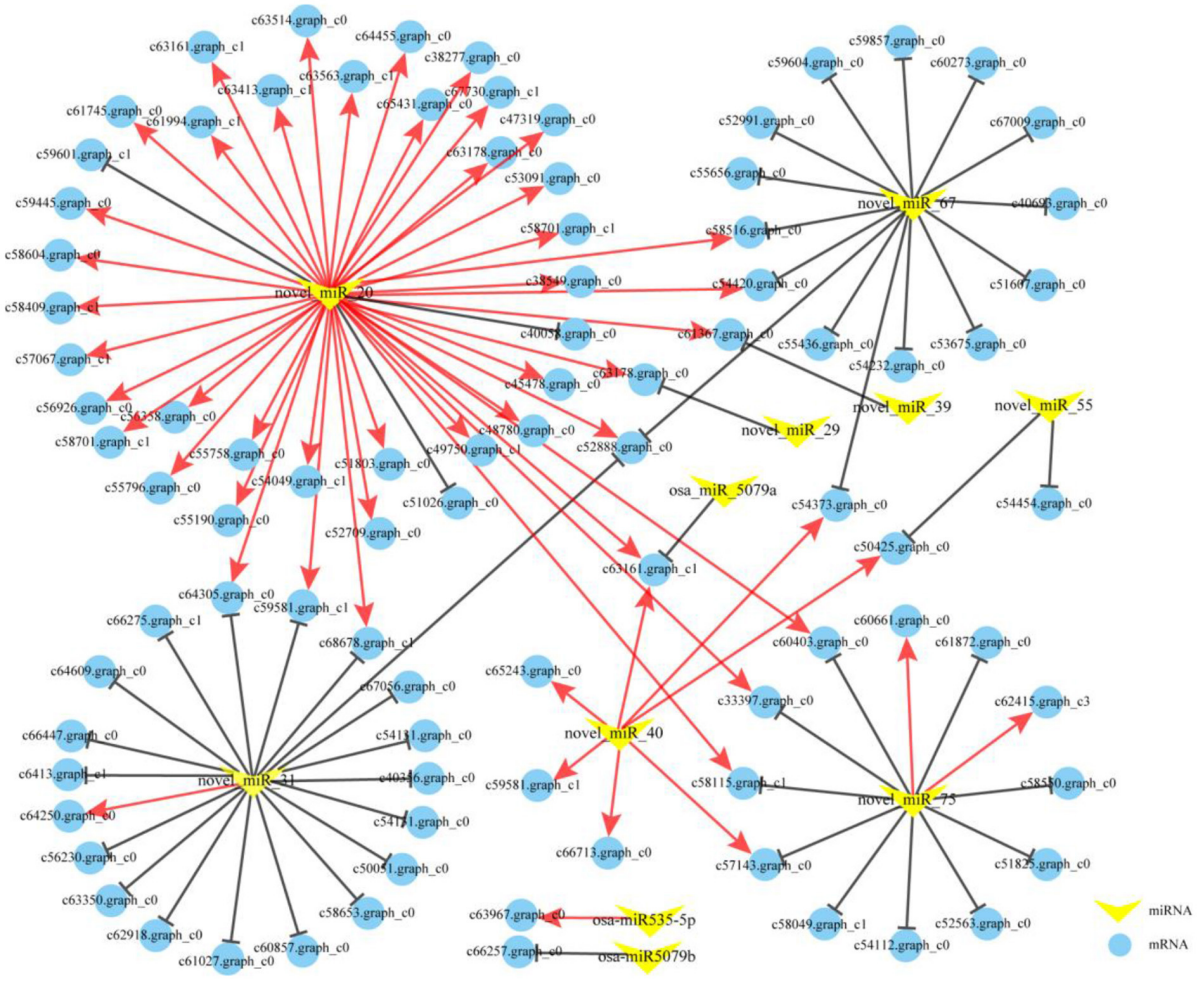
The miRNA-mRNA interaction networks in the greening process of *P. vivax*. The networks were formed from 103 miRNA-mRNA pairs comprising, 11 DEMs (yellow V-shape), and 83 DEGs (blue circle). The red and black solid lines respectively, indicate coherent and non-coherent miRNA-mRNA pairs *via in silico* and degradome analyses.

According to the identified miRNAs and the annotated mRNA sequences of *P. vivax*, a total of 11,851 miRNA-gene interactions, consisting of 115 miRNAs and 8,736 genes, were identified using the TargetFinder software. We observed that 22 DEMs targeted 7,299 genes, and 7,888 miRNA-gene interactions were identified *in silico*. However, 22 DEMs may play important roles in the greening process of *P. vivax*. Further investigation revealed 81 DEM-DEG interactions between the seven DEMs and 71 DEGs (Figure 8). Moreover, 2.62 million clean tags were obtained from degradome sequencing and used to identify the cleavage sites. The results indicated that 670 genes targeted by 87 miRNAs constituted 799 miRNA-target pairs, which were presented in the form of target plots. The target genes of these DEMs were further screened according to the results of *in silico* and degradome analyses. Five predicted DEM-DEG interactions were verified in *in silico* using degradome analyses (Supplementary Figures S3A–E). Additionally, 22 extra DEM-DEG interaction pairs were identified between eight DEMs and 16 DEGs by degradome analysis (Supplementary Table S8). Thus, there were 103 DEM-DEG interactions in the two comparisons (Figure 8).

Interactions between miRNAs and mRNAs can be divided into coherent (positive correlation: opposite expression pattern of miRNA and target mRNA) or non-coherent (negative correlation: similar expression pattern of miRNA and target mRNA) pairs based on their expression patterns (Hausser and Zavolan, 2014). Of the 103 DEM-DEG pairs, 51 were coherent and 52 were non-coherent (Figure 8). Further analysis showed that the 51 coherent pairs consisted of five miRNAs and 49 genes, and 52 non-coherent pairs included 11 miRNAs and 50 genes. These findings indicated that a single miRNA can cleave multiple targets. Interestingly, 40 coherent and three non-coherent pairs interacted with novel_miR_20, and seven coherent pairs were interacted with novel_miR_40, indicating that they might play important roles in the greening process of *P. vivax*. To further investigate the potential functions of the DEMs involved in the greening process of *P. vivax*, the functional enrichment analysis of the 83 target genes was performed using GO and KEGG annotations (Supplementary Figure S4; Supplementary Table S9). GO enrichment analysis showed that the target genes were uniformly assigned to photosynthesis I and photosystem (GO: 0009522 and GO: 0009521), photosynthesis (GO: 0015979), chlorophyll and pigment binding (GO: 0016168 and GO: 0031409). KEGG annotation was performed to explore the pathways in which the identified DEM targets were involved. Eleven pathways were identified, one of which was the metabolic pathway, and the photosynthesis-antenna proteins, flavonoid biosynthesis, and photosynthesis pathways were also enriched.

### Construction and validation of a critical “miRNA-mRNA” regulatory module

**Figure 9:**
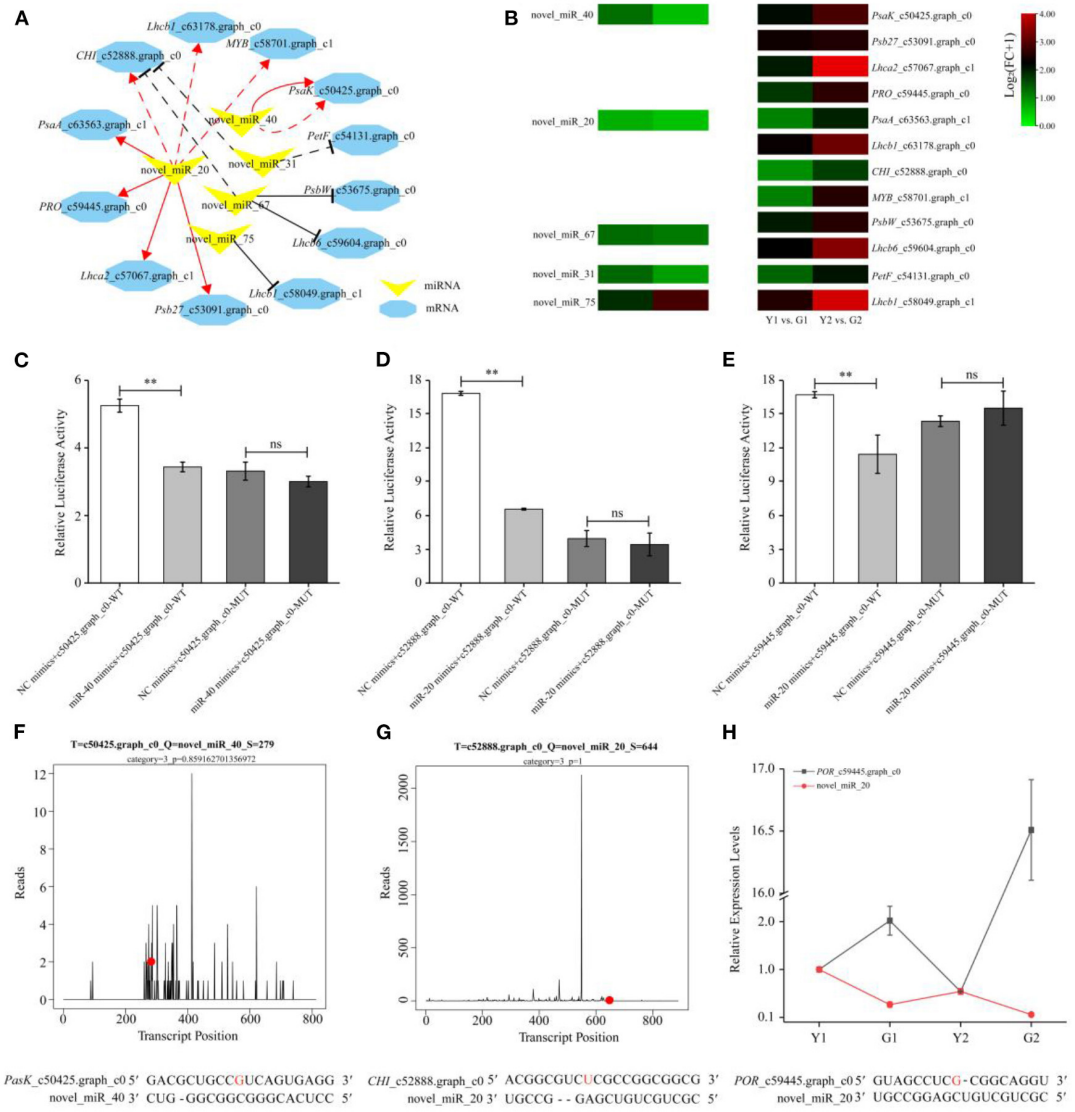
Expression profile and validation of key miRNA-mRNA interaction pairs. **(A)** A representative regulation module related to photosynthesis and pigment metabolism formed by five DEMs (yellow V-shape) and 12 DEGs (blue hexagon). The red solid and dashed lines indicate coherent miRNA-mRNA pairs *via in silico* and degradome analyses, respectively. The black solid and dashed lines indicate non-coherent miRNA-mRNA pairs *via in silico* and degradome analyses, respectively. **(B)** Expression heatmaps of selected pairs of DEMs and their target DEGs. The heatmap on left and right respectively, indicated the expression of DEMs and DEGs. **(C–E)** Verification of three miRNA-mRNA correlation pairs using a dual-luciferase reporter assay. Data is presented as the mean ± standard deviation. Statistical analyses were performed by student's *t*-test, ^**^: *p* < 0.01 and ns: *p* > 0.05. **(F, G)** Nucleotide cleavage positions of novel_miR_40-*PasK*_c50425.graph_c0, and novel_miR_20-*CHI*_c52888.graph_c0 pairs were validated by degradome sequencing. The red dots represent the cleavage nucleotide positions. **(H)** Expression analysis of novel_miR_20-*PRO*_c59445.graph_c0 pair by qPCR.

A regulatory module containing 14 miRNA-mRNA interaction pairs related to photosynthesis and pigment metabolism was constructed using the coherent and non-coherent pairs (Figure 9A). Three non-coherent and four coherent pairs were verified using degradome analyses (Supplementary Figures S3E–I, Figures 9F,G). It was observed that novel_miR_20 and novel_miR_40 were down-regulated, and their corresponding targets were up-regulated during the greening process of *P. vivax*, such as *CHI, PRO, Psb27*, and *PsaK*. Conversely, novel_miR_31, novel_miR_67, and novel_miR_75 were up-regulated in the Y1 vs. G1 comparison, as were their corresponding target genes (Figure 9B). Furthermore, a MYB-related gene family member, *MYB*_c58701.graph_c1 was degraded by novel_miR_20, which showed similar expression patterns to the three genes (correlation ≥ 0.8 and *P* ≤ 0.01) (Supplementary Table S10), indicating that novel_miR_20 might have an influence on the photosynthesis processes by targeting *MYB*_c58701.graph_c1.

Based on the regulatory modules and expression levels of miRNAs and mRNAs, two highly expressed miRNAs (novel_miR_40 and novel_miR_20) and their target genes (*PsaK*_c50425.graph_c0, *CHI*_c52888.graph_c0, and *PRO*_c59445.graph_c0) were validated using qPCR and dual-luciferase reporter assays. qPCR of miRNA-mRNA pairs further validated the reliable results of high-throughput sequencing (Figure 9H, Supplementary Figure S5). For example, the expression of novel_miR_40 was down-regulated, but that of *PsaK*_c50425.graph_c0 was up-regulated in G2 compared to Y2. Meanwhile, degradome and dual-luciferase reporter assays demonstrated that novel_miR_40 could specifically bind to *PsaK*_c50425.graph_c0, and novel_miR_20 could specifically bind to *CHI*_c52888.graph_c0 and *PRO*_c59445.graph_c0 (Figures 9C–G). Luciferase activities of the novel_miR_40 and novel_miR_20 mimics+mRNA-WT groups were all lower than those of the NC mimics+mRNA-WT group (*p* < 0.01) in the transfected cells; however, there was no significant difference between two the MUT groups. These results provide evidence for elucidating the relationship between genetic elements in the miRNA-mRNA regulatory module.

## Discussion

### De-domestication influenced phenotypic characters and photosynthetic efficiency of DGM

De-domestication is an interesting phenomenon for plants. In this study, compared with CYP, DGM displayed a better-adapted phenotype, such as larger DBH and IWT, as well as larger ID of portion of internodes (Figures 1A–D). Considering these results, we suggest that greater more biomass accumulation could be attributed to green culms with higher photosynthetic efficiency (Figures 1E,F). The results showed that the chloroplast structure of the green culms of *P. vivax* was more complete than that of yellow culms, and the chlorophyll content of the green culm was significantly higher than that of the yellow culms using LSCM (Xia et al., 2015). Similar results were observed in the present study (Figures 2B,C). Many studies have shown that flavonoids, carotenoids, and anthocyanins may be involved in plant coloration (Hu et al., 2020; Jiang et al., 2020; Wei et al., 2021). In *P. violascens* cv. Viridisulcata, the flavonoid content of yellow stems was significantly higher than that of the green sulcus in the same bamboo culm (Wei et al., 2021). Different results were obtained in this study; the flavonoid and carotenoid contents of DGM were higher than those of CYP (Figures 2D,E), despite the fact that DGM and CYP bamboo were born on the same rhizome. The coloration of culms is a complex process, that might be influenced by many factors, such as the number of pigments, metal ions, or molecular conformations (Jiang et al., 2020).

Photosynthesis is pivotal for plant growth and yield (Yang et al., 2014), and gene encoding photosynthetic enzymes play significant roles in photosynthesis and pigmentation (Pashayeva et al., 2021; Li et al., 2022b). The KEGG results showed that photosynthesis and photosynthesis-antenna proteins were significantly enriched (Figure 3C). It was worth noticing that there were 27 DEGs related to photosynthesis (Figure 4). Research has shown that the *Lhc*s in PSI and PSII play important roles in light-harvesting (Chu et al., 2015). In the current study, all DEGs associated with *Lhc*s, such as *Lhca2, Lhcb1*, and *Lhcb3-6* were up-regulated in DGM culms (Figures 4B,D). Similarly, two members sharing a high amino acid percentage of Lhcs have been identified in *P. vivax* using an SSH cDNA library (Xia et al., 2015). In addition, subunits of PSI and PSII play crucial roles in electron transmission and energy metabolism. For example, Psb27 is essential for the energy metabolism in PSII (Chen et al., 2006). PsbS might stabilize the PSII-LHCII complex structure and improve electron transmission efficiency (Dong et al., 2015). During the light reaction of photosynthesis, genes encoding subunits of PSI and PSII in the DGM were up-regulated (Figures 4C,E), which promoted the absorption and transmission of light energy, ultimately improving the efficiency of light capture. We speculated that these genes played important roles in promoting photosynthesis by increasing the efficiency of light absorption and electronic transmission (Tian et al., 2020).

### The diversity of DEGs involved in chlorophyll and flavonoid metabolism

Chlorophyll is an essential indicator of photosynthetic efficiency and nutritional status, and is also an important pigment for determining the skin color of plants (Wang et al., 2020a). Genes related of chlorophyll synthesis were also identified (Lai et al., 2015; Wen et al., 2015). The functions of enzymes encoded by key genes, such as ChlH, POR and CAO, have been well-studied, (Tanaka and Tanaka, 2006, 2007). The ChlH enzyme catalyzed protoporphyrin IX to form Mg-protoporphyrin IX, which is the first critical step in chlorophyll formation (Wu et al., 2009). The POR was an important enzyme that catalyzed divinyl protochlorophyllide to generate divinyl chlorophyllide a, which is a critical product of the chlorophyll cycle (Kwon et al., 2017). Here, five DEGs were identified in the chlorophyll synthesis pathway (Figure 5), which were up-regulated in DGM compared to CYP, suggesting that they may promote the synthesis of chlorophyll a and chlorophyll b (Figures 2A,B). Similarly, *ChlH, ChlE*, and *POR* have been reported as the key genes involved in the chlorophyll synthesis pathway, which participate in the color formation of cucumber (*Cucumis sativus*) skins (Wang et al., 2020a). CAO has been reported to play an important role in affecting the contents of chlorophyll a and chlorophyll b, thereby influencing broccoli (*Brassica oleracea*) yellowing (Luo et al., 2019). These results suggested that chlorophyll synthesis genes are culm colouration in bamboo.

Flavonoids are widely distributed in plants (Xu et al., 2015), and their content has a crucial effect on plant colors (Vogt, 2010). Flavonoids have been reported participate in the color-change process of red maple (*Acer palmatum* ‘*Atropurpureum*’) leaves and cherry fruits (Lu et al., 2020; Tian et al., 2020). Available evidence indicates that CHS, CHI, F3H, and ANS are essential for flavonoid biosynthesis (Vogt, 2010). A previous study showed that the high transcript abundances of *PvCHS* and *PvCHI* were detected in green sulcus and yellow culm of *P. violascens*, despite the significant difference in flavonoid content (Wei et al., 2021). Compared with the Lv sample, *CHS* was significantly repressed and two metabolites (naringenin chalcone and naringenin) of the Bai sample were also decreased (Wang et al., 2020a). Different results appeared in this study, six DEGs involved in flavonoid biosynthesis were up-regulated in DGM compared to CYP (Figure 6). However, more enzyme activities of CHI and contents of flavonoids were detected in DGM than in CYP (Figures 6G, 2D), but the content of anthocyanin showed an opposite trend (Figure 2G). We speculated that the differences in the samples and detection methods might be the cause of the diversity in the results. In addition, the processes of culm color deposition are complex, and many regulatory factors (TFs and miRNAs) may participate in the flavonoid synthesis pathway (Shi et al., 2021; Lv et al., 2022).

### miRNAs associated with de-domestication of bamboo culms color by regulating pigment biosynthesis and photosynthesis

Numerous studies have shown that miRNAs can affect plant pigment biosynthesis and photosynthesis by regulating target gene expression (Wang et al., 2020b; Yang et al., 2021a; Zhang et al., 2021). Ectopic overexpression of bol-miR171b increased chlorophyll content in broccoli (Li et al., 2018). MiR156 regulates anthocyanin biosynthesis through *SPL* targets in poplars (*Populus alba* × *P. tremula* var. *glandulosa*) (Wang et al., 2020b). In this study, 22 DEMs were identified (Figure 7) and an interaction network containing 103 DEM-DEG interaction pairs was constructed using 11 DEMs and 83 DEGs (Figure 8). These DEGs were annotated in pigment synthesis pathways of photosynthesis and flavonoid metabolism (Supplementary Figure S4). We speculated that miRNAs might regulate the expression of gene, thereby playing an important role in the color formation of bamboo culms (Zhang et al., 2021). It is well-known that, the regulatory mechanisms of miRNAs are conserved, and the functions of miRNAs are multiple. Studies have shown that some miRNAs can function together in the same pathway. For example, novel_miR_20 and novel_miR_40 target photosynthesis pathway genes (Figure 8). Similar results were found in poplars, with two miRNAs targeting *VDE* acting on the carotenoid synthesis pathway (Wang et al., 2020b). However, the same miRNA may act *via* different pathways or different steps of the same pathway (Li et al., 2020a,b). Novel_miR_20 targeted *PRO* and *CHI* to regulate the chlorophyll and flavonoid biosynthesis, respectively (Figure 8). Therefore, miRNAs and their targets form intricate regulatory networks.

**Figure 10:**
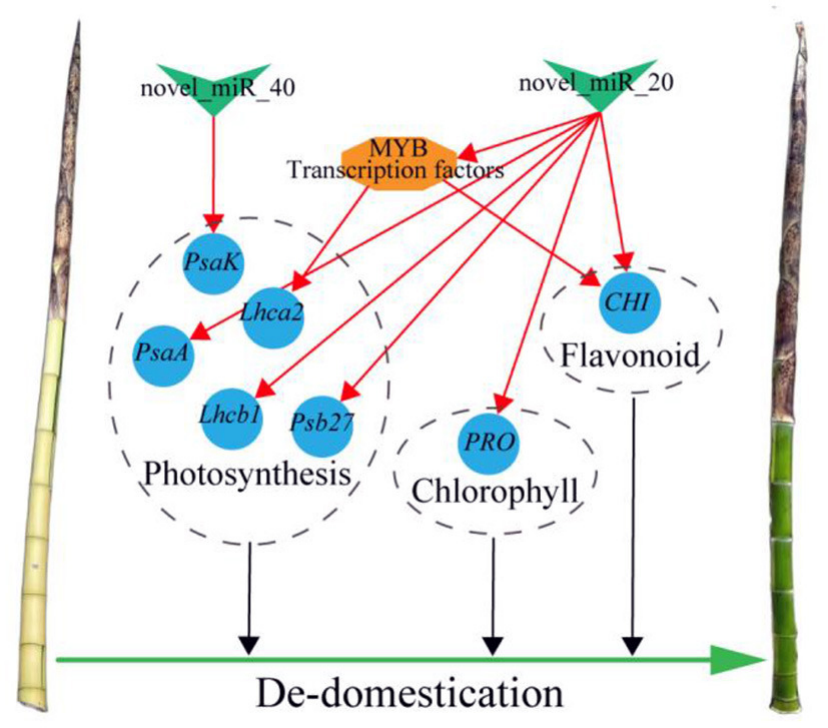
Summary figure of the mechanism of de-domestication in regulating culm color of *P*. *vivax* cv. Green V-shape, blue circle, and orange hexagon respectively, indicate miRNAs, structural genes, and TF. The red lines indicate coherent miRNA-mRNA or TF-mRNA pairs.

In this network, eight coherent miRNA-mRNA pairs regulated a complicated pigment biosynthesis process, revealing a possible miRNA-mediated mechanism of pigment biosynthesis in *P. vivax* (Figure 9A). Furthermore, our comprehensive analysis of miRNA function in pigment biosynthesis and photosynthesis regulatory network suggested that novel_miR_40-*PasK*_c50425.graph_c0, novel_miR_20-*CHI*_c52888.graph_c0, and novel_miR_20-*PRO*_c59445.graph_c0 pairs might constitute the core module in regulating culms discoloration (Figure 9A). Many “miRNA-mRNA” modules are closely related to color sedimentation in plants (Wang et al., 2020b; Yang et al., 2021a). Thus, the three “miRNA-mRNA” modules were validated by qPCR and dual-luciferase reporter assays in this study (Figures 9C–H). In conclusion, we consider that miRNAs could play important roles in pigment biosynthesis in *P. vivax*, thus affecting the photosynthetic efficiency of culms (Figure 10). However, it is difficult to conduct further functional verification because of the lack of genomic information and genetic transformation systems of *P. vivax*. Our study is the first to reveal that miRNAs may function as new regulators of color variation in *P. vivax* culms, which is associated with de-domestication of bamboo.

## Conclusion

De-domestication can provide valuable traits and genetic resources for modern crop breeding. In this study, the de-domesticated bamboo culms had larger diameter at breast height and internode diameter, thicker internode wall, higher net photosynthetic rate and stomatal conductance, higher contents of chlorophyll, flavonoid carotenoid and soluble sugar, and lower contents of anthocyanin content than the domesticated bamboo culms. A total of 295 DEGs were detected in the two different colored bamboo culms, of which 27 were related to photosynthesis and 11 were associated with chlorophyll metabolism and flavonoid biosynthesis pathways. Furthermore, 22 DEMs were identified in this study. A regulatory network of 103 miRNA-mRNA pairs was established by integrated analysis of mRNAs and miRNAs, among which five DEM-DEG interactions predicted *via in silico* analysis were verified by degradome analyses. Furthermore, a representative regulation module related to photosynthesis and pigment metabolism, which consisted of five DEMs and 12 DEGs, was validated by qPCR and a dual-luciferase reporter assay. Our study provides new insights into the molecular mechanism of *P. vivax* culm color formation and reveals that miRNAs might function in the de-domestication of culm color variation in *P. vivax* at the first time.

## Data availability statement

The datasets presented in this study can be found in online repositories. The names of the repository/repositories and accession number(s) can be found below: https://www.ncbi.nlm.nih.gov/, PRJNA839516; https://www.ncbi.nlm.nih.gov/, PRJNA841040.

## Author contributions

ZG designed and supervised this study. CZ and YLou collected all the data. CZ, KY, and YLiu performed the data analysis. CZ and ZG prepared the manuscript. CZ, XX, DG, ZL, HS, and ZG modified the manuscript. All authors contributed to the article and approved the submitted version.

## Funding

This research was supported by the National Key Research and Development Program of China (Grant No. 2021YFD2200502), the National Natural Science Foundation of China (Grant No. 31971736).

## Conflict of interest

The authors declare that the research was conducted in the absence of any commercial or financial relationships that could be construed as a potential conflict of interest.

## Publisher's note

All claims expressed in this article are solely those of the authors and do not necessarily represent those of their affiliated organizations, or those of the publisher, the editors and the reviewers. Any product that may be evaluated in this article, or claim that may be made by its manufacturer, is not guaranteed or endorsed by the publisher.

## Supplementary material

Supplementary Figure S1 Characteristics of three parameters of CYP and DGM culms.

Supplementary Figure S2 PCA analysis of four samples.

Supplementary Figure S3 Nucleotide cleavage position of miRNAs and the target genes validated by degradome sequencing.

Supplementary Figure S4 GO and KEGG analyses of 83 target DEGs.

Supplementary Figure S5 Expression analysis of two miRNA-mRNA pairs by qPCR.

Supplementary Table S1 The detailed information of transcriptome and small RNA data.

Supplementary Table S2 The primer sequences used in this study.

Supplementary Table S3 Sequences of NC and miRNA mimics, as well as WT and MUT in plasmids.

Supplementary Table S4 Transcriptome sequencing information of 12 samples.

Supplementary Table S5 Unigene information of transcriptome sequencing.

Supplementary Table S6 Unigene annotations based on nine databases.

Supplementary Table S7 MiRNA members in different miRNA families.

Supplementary Table S8 Detailed information of 25 DEM-DEG pairs by eight miRNAs and 16 genes.

Supplementary Table S9 Annotations of 83 target gene based on nine databases.

Supplementary Table S10 Correlation analysis of *MYB*_c58701.graph_c0 and 11 genes.
