# Supplementary material for: Integrative analyses of morphology, physiology, and transcriptional expression profiling reveal miRNAs involved in culm color in bamboo
Source: Front Plant Sci. 2022 Sep 9;13:992794. doi: 10.3389/fpls.2022.992794 (PMC9508110; doi:10.3389/fpls.2022.992794)
Supplement: Supplementary Figure S1 — Characteristics of three parameters of CYP and DGM culms. [file Data_Sheet_1.docx]

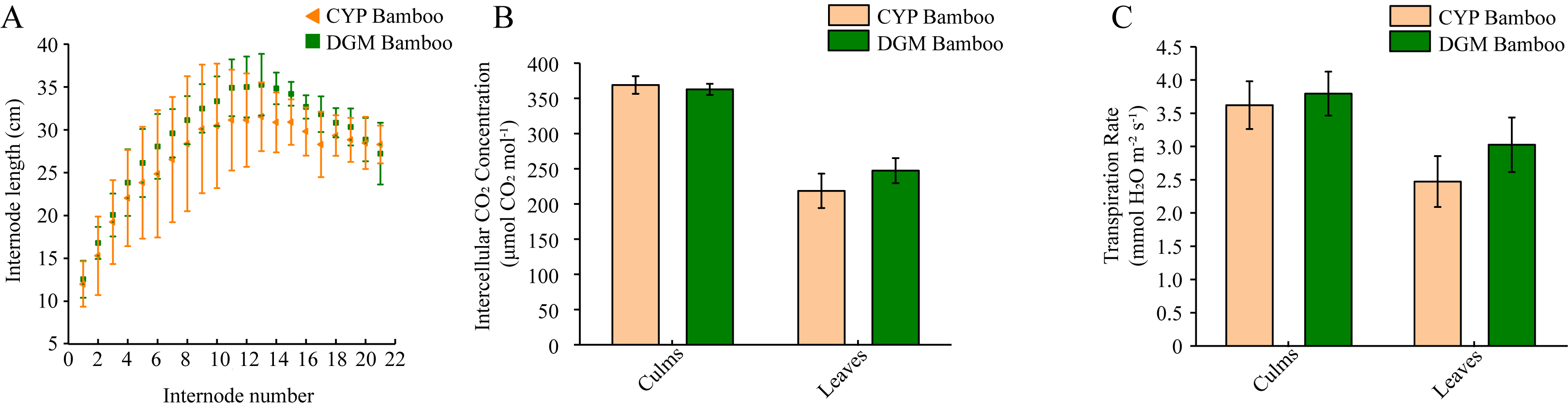


Figure S1 Characteristics of three parameters of CYP and DGM culms. (A) Internode length of CYP and DGM culms. (B) Intercellular CO_2_ concentrations of culms and leaves of CYP and DGM. (C) A transpiration rates of culms and leaves of CYP and DGM. Error bars indicate standard deviation (SD) of three technical repeats.


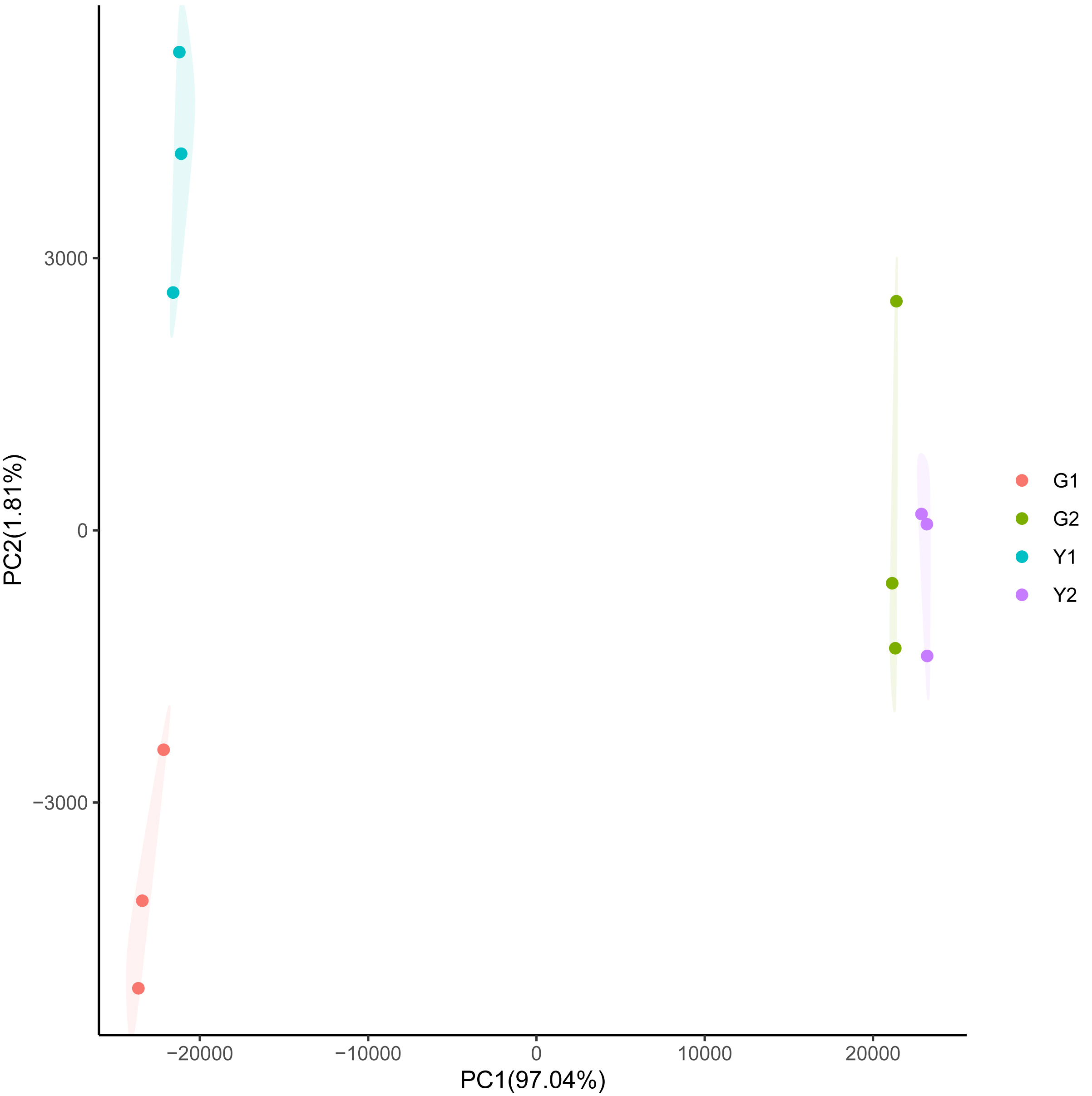


Figure S2 PCA analysis of four samples. The red, blue, green, and purple circles indicate G1, Y1, G2 and Y2 samples, respectively.
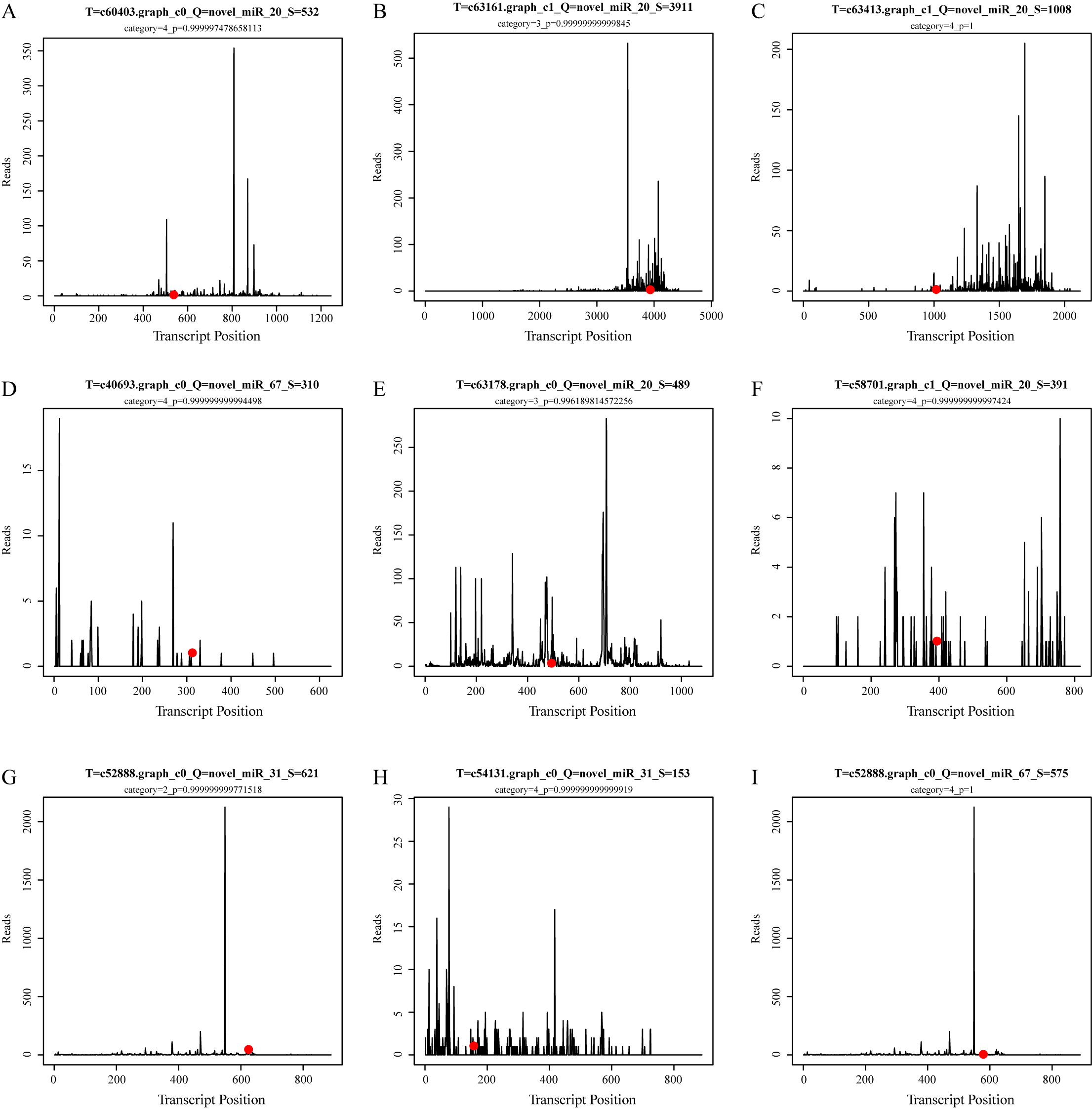


Figure S3 Nucleotide cleavage position of miRNAs and the target genes validated by degradome sequencing. The red dots represent the cleavage nucleotide positions.


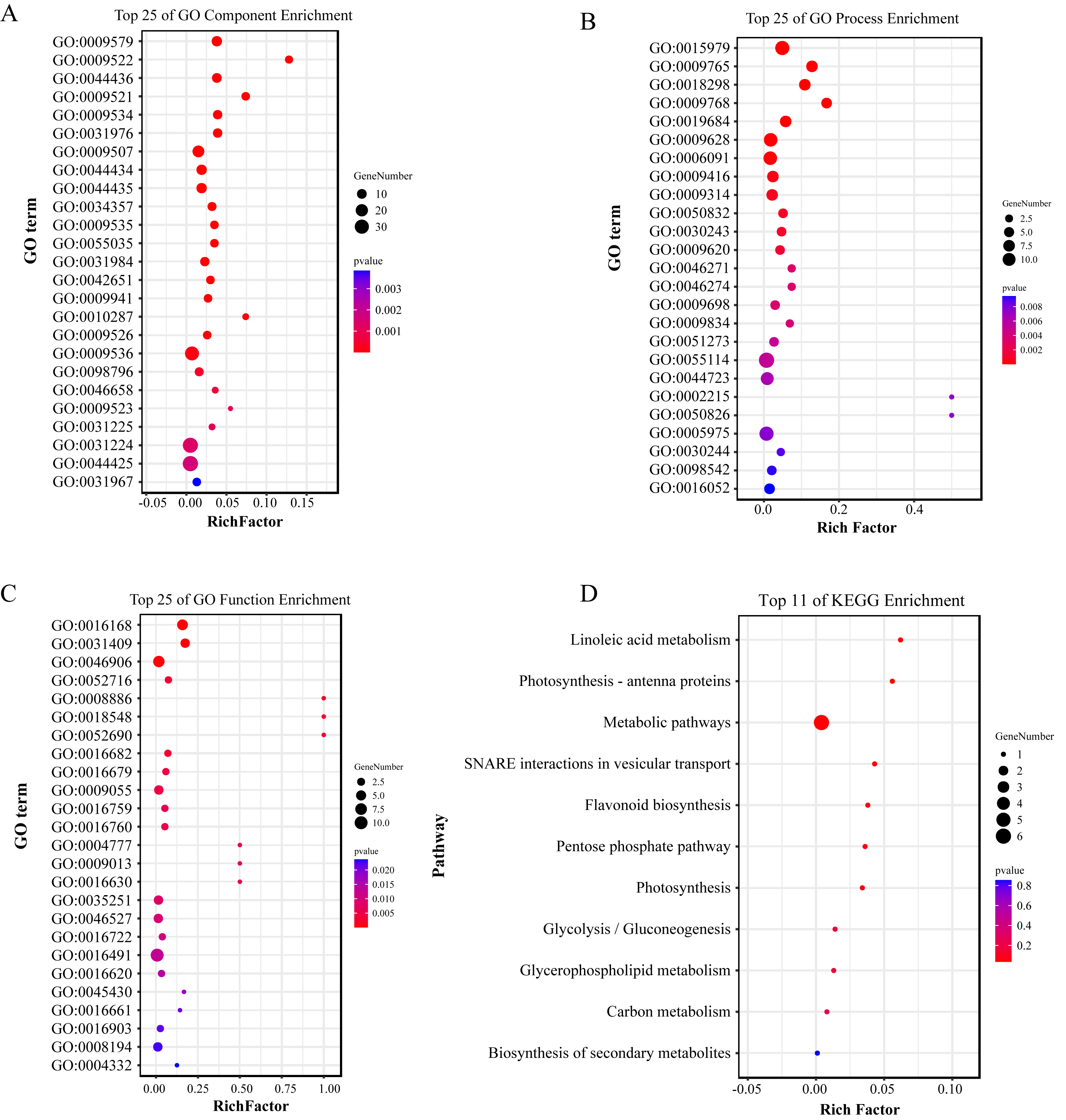


Figure S4 GO and KEGG analyses of 83 target DEGs. (A-C) GO term enrichment analysis, including cellular component (A), biological process (B), and molecular function (C). (D) KEGG enrichment analysis. The size of the circles indicates the enriched genes number. The color of the circle indicates the p-value of enrichment.


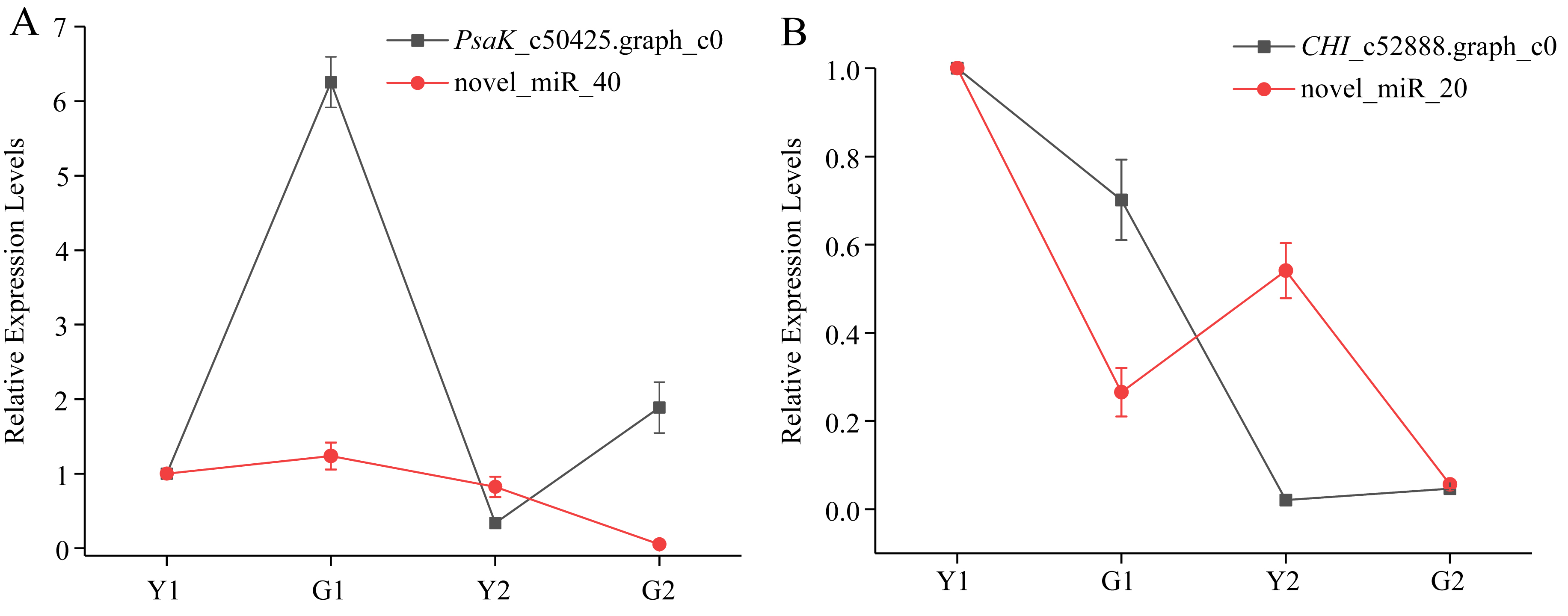


Figure S5 Expression analysis of two miRNA-mRNA pairs by qPCR. (A) Expression analysis of novel_miR_40-*PsaK*_c50425.graph_c0 pair, and (B) novel_miR_20-*CHI*_c52888.graph_c0 pair. Y1 and G1 indicate the 7^th^ internodes with yellow or green color, Y2 and G2 indicate the 11^th^ internodes with yellow or green color, respectively. Error bars indicate standard deviation (SD) of three technical repeats.
